# Functional Characterization of Carbohydrate-Binding Modules in a New Alginate Lyase, TsAly7B, from *Thalassomonas* sp. LD5

**DOI:** 10.3390/md18010025

**Published:** 2019-12-26

**Authors:** Zhelun Zhang, Luyao Tang, Mengmeng Bao, Zhigang Liu, Wengong Yu, Feng Han

**Affiliations:** 1 Key Laboratory of Marine Drugs (Ministry of Education), Shandong Provincial Key Laboratory of Glycoscience and Glycoengineering, School of Medicine and Pharmacy, Ocean University of China, Qingdao 266003, China; popxzxz@163.com (Z.Z.); tlytwzwy@163.com (L.T.); baomengmengsdu@163.com (M.B.); liuzhigangzzy@163.com (Z.L.); 2 Laboratory for Marine Drugs and Bioproducts of Qingdao National Laboratory for Marine Science and Technology, Qingdao 266237, China

**Keywords:** alginate lyase, carbohydrate-binding module, enzymatic characterization, thermostability, enzymatic activity, product distribution, polysaccharide lyase, brown algae

## Abstract

Alginate lyases degrade alginate into oligosaccharides, of which the biological activities have vital roles in various fields. Some alginate lyases contain one or more carbohydrate-binding modules (CBMs), which assist the function of the catalytic modules. However, the precise function of CBMs in alginate lyases has yet to be fully elucidated. We have identified a new multi-domain alginate lyase, TsAly7B, in the marine bacterium *Thalassomonas* sp. LD5. This novel lyase contains an N-terminal CBM9, an internal CBM32, and a C-terminal polysaccharide lyase family 7 (PL7) catalytic module. To investigate the specific function of each of these CBMs, we expressed and characterized the full-length TsAly7B and three truncated mutants: TM1 (CBM32-PL7), TM2 (CBM9-PL7), and TM3 (PL7 catalytic module). CBM9 and CBM32 could enhance the degradation of alginate. Notably, the specific activity of TM2 was 7.6-fold higher than that of TM3. CBM32 enhanced the resistance of the catalytic module to high temperatures. In addition, a combination of CBM9 and CBM32 showed enhanced thermostability when incubated at 80 °C for 1 h. This is the first report that finds CBM9 can significantly improve the ability of enzyme degradation. Our findings provide new insight into the interrelationships of tandem CBMs and alginate lyases and other polysaccharide-degrading enzymes, which may inspire CBM fusion strategies.

## 1. Introduction

Brown algae are composed of mannitol, alginate, cellulose, fatty acids, and inorganic salts, and represent a key raw material for the production of biofuels. Mannitol and alginate, which are the main components of brown algae, serve as the main convertible carbohydrates. The proportion of alginate in brown algae can reach up to 40% of the dry weight [1]. Alginate is an acidic heteropolysaccharide and can be classified into three different types according to its β-d-mannuronate (M) and α-l-guluronate (G) content: polyM, polyG, and alternating or random polyMG blocks [2]. Owing to its high viscosity and gelling properties, alginate is widely used in the food and pharmaceutical industries [3].

Alginate lyases belong to the family of polysaccharide lyases (PLs) and catalyze the degradation of 1,4-glycosidic bonds via β-elimination to form a double bond between C-4 and C-5 at the nonreducing end [4]. The alginate oligosaccharide end products of the majority of alginate lyases have a wide range of applications. This versatility can be attributed to their antioxidant [5,6], anti-hypersensitive [7,8], anti-proliferative [9], and anti-allergic properties [10]; they have also been shown to promote plant growth [11].

Several thousand alginate lyases have been classified into the PL5, 6, 7, 14, 15, 17, 18, 32, 34, 36 and 39 families in the CAZy database (http://www.cazy.org/) [4,12]. Some alginate lyases are known to contain one or more carbohydrate-binding modules (CBMs); CBM13, CBM16, and CBM32 are the most common [13]. CBMs have a wide range of applications in biomedicine, molecular biology, and the paper, food, and textile industries [14]. Previous research has demonstrated that CBMs can recognize a range of crystalline, amorphous, soluble and non-soluble polysaccharides in some carbohydrate-active enzymes via substrate-specific affinity [15]. In the degradation of cellulosic substrates, CBMs have been shown to work synergistically and to produce cumulative effects [16]. In alginate lyase, CBM32s have been elucidated to participate in the recognition of alginate and impact the composition of final degradation products, which provided potential and valuable application and aroused wide interests in function of CBM [17,18]. However, the precise function of CBM9 in alginate lyases has yet to be elucidated.

In this study, we investigated the function of two CBMs, CBM9 and CBM32, in an alginate lyase, TsAly7B, from the marine bacterium *Thalassomonas* sp. LD5. This enzyme contains an N-terminal CBM9 module followed by a CBM32 module and a C-terminal PL7 catalytic module. We cloned and expressed the full-length (FL) TsAly7B and a series of truncated mutants, TM1 (CBM32-PL7), TM2 (CBM9-PL7), and TM3 (PL7 catalytic module). Our analysis showed that CBM9 and CBM32 have key roles in the degradation of alginate and in the thermostability of TsAly7B. This description of the functional activities of the different modules in TsAly7B is expected to provide insights into the relationship between the two CBMs in this alginate lyase.

## 2. Results

### 2.1. Cloning and Sequence Analysis of TsAly7B

**Figure 1:**
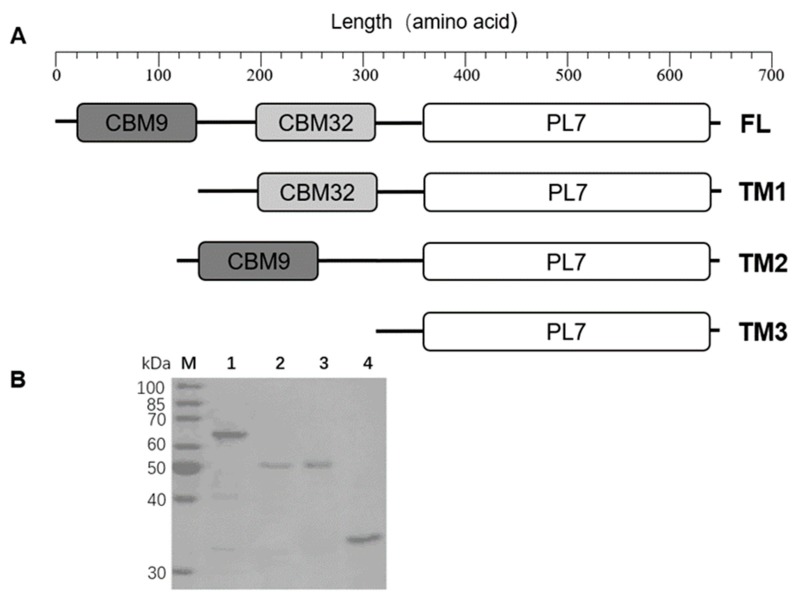
Construction of FL TsAly7B and three truncated mutants. (**A**) Domain structure of FL TsAly7B and three truncated mutants. (**B**) SDS_PAGE showing the FL, TM1, TM2, and TM3 proteins. Lane M, marker. Lane 1, TsAly7B-FL. Lane 2, TM1. Lane 3, TM2. Lane 4, TM3.

In a previous study, a new marine bacterium, *Thalassomonas* sp. LD5, that was capable of degrading alginate was isolated and identified in our laboratory [19]. By analyzing the genomic sequence of this bacterium, we predicted five alginate lyase genes encoding one form of PL6 (TsAly6A) [19], three forms of PL7 (TsAly7A, TsAly7B, TsAly7C), and one form of PL17 lyase (TsAly17A). We found that the *Tsaly7B* gene was 1827 bp in length and encoded a multi-domain protein of 608 amino acids (Figure 1A). The TsAly7B protein had a predicted molecular mass of 64.78 kDa and an estimated isoelectric point (pI) of 4.73. SignalP 5.0 software predicted an N-terminal 24 amino acid signal peptide.

CD-searches identified a CBM_4_9 superfamily module (Ile29-Tyr130) at the N-terminus, followed by an F5_F8_type_C module (His184-Glu296) and a catalytic module (Phe338–His603) belonging to the PL7 family at the C-terminus. Phylogenetic tree analysis of CBM_4_9 and F5_F8_type_C showed that the CBM_4_9 in TsAly7B could be classified into the CBM9 family, and the F5_F8_type_C in TsAly7B can be classified into the CBM32 family. The FL TsAly7B protein showed the highest identity (60%) with the cyclic nucleotide-binding protein from *Shewanella* sp. P1-14-1. Two CBMs, CBM9 and CBM32, showed the highest identities with one hypothetical protein (65%) from *Vibrio halioticoli* (GenBank accession number: WP_023404110.1) and a hypothetical protein (61%) from *Saccharophagus degradans* (GenBank accession number: WP_011469743.1), respectively. The catalytic module of TsAly7B was most homologous to the alginate lyase AlyB, which features an F5_F8_type_C domain and a PL7 catalytic domain from *Vibrio spendidus* OU02, with 46% identity across all characterized enzymes. 

### 2.2. Expression and Purification of Recombinant TsAly7B and Three Truncated Mutants

To investigate the function of CBM9 and CBM32 in TsAly7B, we constructed recombinant plasmids encoding the FL protein and three TMs in which we deleted one or two of the CBMs (TM1, TM2, and TM3) (Figure 1A). To characterize these recombinant proteins, we used the *Escherichia coli* BL21(DE3)/pET-28a (+) system to express the FL TsAly7B and three TMs. Target proteins were purified by Ni^2+^ affinity chromatography using different concentrations of imidazole. SDS-PAGE analysis of the purified proteins showed single bands at 65, 51, 53 and 34 kDa, respectively (Figure 1B). The molecular masses of these recombinant proteins were consistent with their theoretical molecular masses (64.5, 51.3, 52.6 and 33.8 kDa, respectively). 

### 2.3. Biochemical Characterization of TsAly7B and Three Truncated Mutants

**Figure 2:**
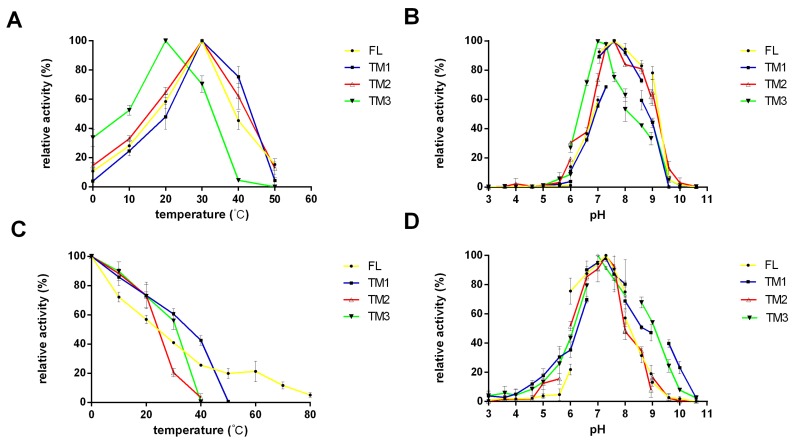
Effects of temperature and pH on the FL TsAly7B and three truncated mutants. Optimal temperature (**A**), optimal pH (**B**), thermal stability (**C**), and pH stability (**D**) of the FL TsAly7B and three truncated mutants. For optimal temperature (**A**) and optimal pH (**B**), the highest activity of each enzyme (11.7 U/mL) was set as 100%; for thermal stability (**C**), and pH stability (**D**), the original activity (15.1 U/mL) was set as 100%. Values represent the mean of three replicates ± standard deviation.

The optimal temperature for enzymatic activity was 30 °C for FL, TM1, and TM2 but 20 °C for TM3 (Figure 2A). We found that TM1 showed the highest thermostability of all four recombinant proteins, with an enzymatic activity of 60% after storage at 30 °C for 1 h; by contrast, the TM2 protein was unstable under such conditions and retained only 20% activity (Figure 2C). Interestingly, the FL enzyme retained approximately 20% enzymatic activity after incubation at 60 °C for 1 h, and 6% after incubation at 80°C. These data suggested that the co-existence of CBM9 and CBM32 could increase the thermal stability of the FL recombinant protein at high temperatures. 

To identify the optimal pH reaction conditions, we set up reaction systems using different buffers with pH values ranging from 3.0 to 10.6. The optimal pH was 7.6 for FL, TM1, and TM2, but 7.3 for TM3 (Figure 2B). These results showed that neither of the CBMs had any significant effect on the optimal pH of the enzyme. The FL enzyme showed 78% enzyme activity at pH 9.0; by contrast, TM1 and TM2 exhibited only 60% enzyme activity at pH 9.0. Furthermore, when we measured enzyme activity in highly alkaline environments (pH > 8.6), we found that FL and TM2 exhibited higher activity than TM1 and TM3 (Figure 2B). When FL, TM1, and TM2 were stored in phosphate buffer at pH 7.3, only a minimal loss of enzymatic activity was observed (Figure 2D); similar results were observed when TM3 was stored at pH 7.0. Notably, TM1 retained the most enzymatic activity when placed in either acidic (pH 3–5.6) or alkaline (pH 9.6–11) environments.

**Figure 3:**
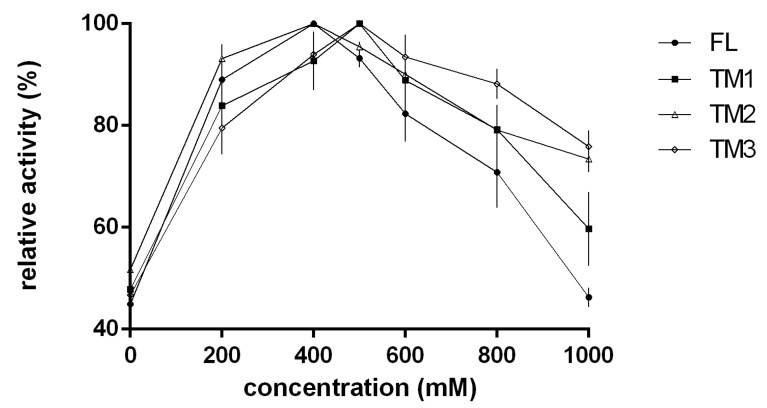
Effects of NaCl concentration on the activity of the FL TsAly7B and three truncated mutants. The relative activity of 100% was determined at the optimal pH, temperature, and NaCl concentration.

Considering the significance of NaCl for marine enzymes, we measured enzyme activity in the presence of different NaCl concentrations. Results showed that the addition of NaCl (0–1 M) to the reaction enhanced the activity of TsAly7B and the three TMs (Figure 3). The optimal NaCl concentration for TM1 and TM3 was 500 mM, while that for FL and TM2 was 400 mM. The differences between the mutants and FL were unremarkable, thus showing that the impact of NaCl concentration was relevant only to the catalytic module. The effect of metal ions on the activity of each recombinant enzyme is shown in Table 1. The addition of Ca^2+^ into the reaction system led to a slight increase (14%) in the activity of the FL protein compared with that observed without metal ions. Ba^2+^ could inhibit the activity of TM2 by 50%, and Mn^2+^ could inhibit the activity of TM3 by 50%. In addition, all of the metal ions led to lower enzyme activities of the TM3 protein compared with the FL protein. These results demonstrate that CBM9 and CBM32 improved the resistance of the catalytic module against metal ions.

The specific activity of TM2 was the highest (60,400 U/μmol) of all the recombinant proteins, while the specific activity of the catalytic module was the lowest (Table 2). The presence of CBM32 also improved the specific activity.

### 2.4. Analysis of the Substrate Specificity and Final Reaction Products

**Figure 4:**
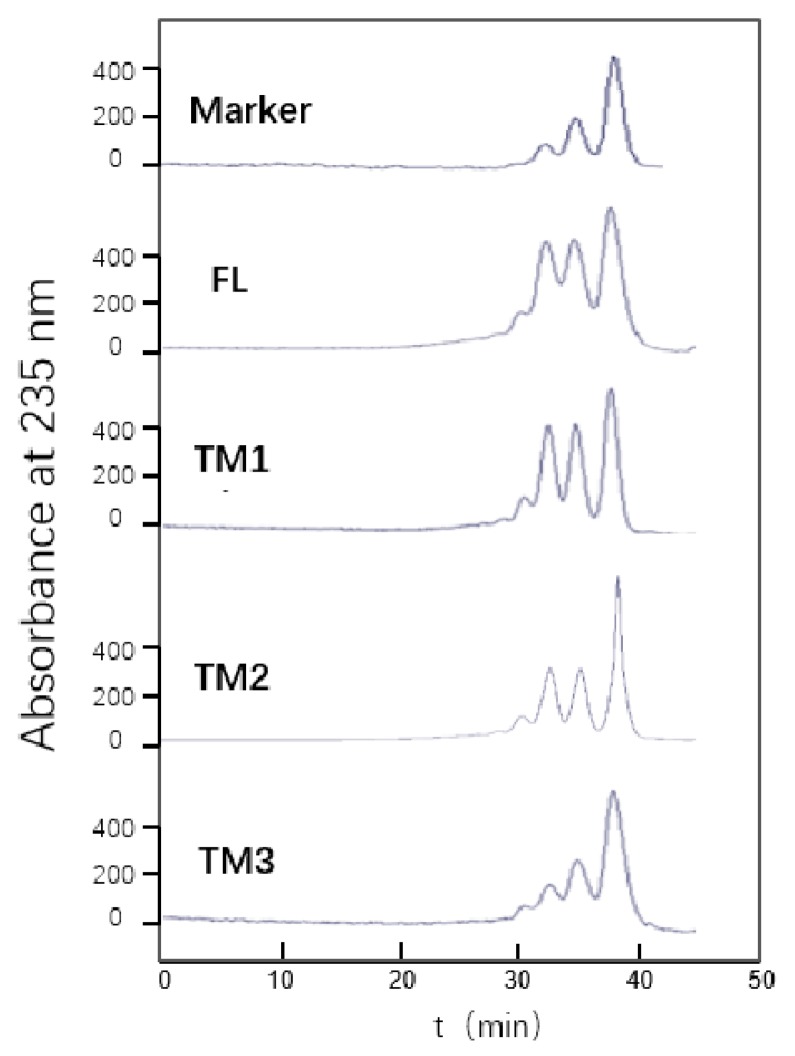
FPLC analysis of end products of the FL TsAly7B and three truncated mutants. Unsaturated disaccharides, trisaccharides, and tetrasaccharides were eluted at 34.2, 32.4, and 30.8 min, respectively. The mixture of unsaturated disaccharides, trisaccharides, and tetrasaccharides was used as the marker, and the total uronic acid concentration was 0.2% (*w/v*).

Next, we investigated the effects of the two CBMs on enzymatic activity by analyzing the substrate specificity and final products of TsAly7B and the three TMs (Table 3). We found that TM3 showed higher activity against polyM than polyG; all three of the other recombinant proteins exhibited similar activities towards polyM and polyG. The final degradation products from reactions involving the FL, TM, and TM2 recombinant proteins were all disaccharides, trisaccharides, and tetrasaccharides; the common ratio for these products was approximately 1.1:1:1. The final degradation product of TM3 was characteristic in terms of the relative proportions of disaccharides, trisaccharides, and tetrasaccharides, with a ratio of approximately 4:2:1. We did not observe any cumulative effects or synergistic effects, although we were able to ascertain that CBMs affected the degree of polymerization (DP) of the final products (Figure 4). Since there is no monosaccharide in the end products, the FL, TM, TM2 and TM3 degraded alginate in an endolytic manner.

## 3. Discussion

Genome annotation identified five alginate lyases in the marine bacterium *Thalassomonas* sp. LD5 from coastal sediments on the Yellow Sea shore of China. Analysis of these alginate lyases showed that TsAly7B could be classified as a member of PL7 family, and that it had two CBMs (CBM9 and CBM32), which is not common in alginate lyases. The function of these CBMs has not been fully characterized. We cloned and expressed TsAly7B and three truncated mutants to study the function of CBM9 and CBM32 in alginate lyase TsAly7B.

A previous study showed that CBM9 enhanced the thermostability and catalytic activity of the catalytic module during the degradation of xylans, and CBM9 had a positive effect on enzymatic activity in the alkaline environment compared with a single catalytic module [20]. Another study described how CBM_4_9 facilitated the hydrolytic properties of GH10 catalytic modules [21]. The CBM32 did not contribute to enhancing the enzymatic activity of AlyB from *Vibrio spendidus* OU02 but served as a “pivot point” during the trisaccharide release process [18]. The presence of CBM32 domain in AlyQ from *Persicobacter* sp. CCB-QB2 also did not significantly increase its activity [17]. In this study, we observed that the specific enzymatic activities of TM1 (CBM32-PL7) and TM2 (CBM9-PL7) were 4.6-fold and 6.6-fold higher than that of TM3 (the PL7 catalytic module only), respectively. This result showed that both CBM9 and CBM32 could improve the activity of PL7 catalytic module of TsAly7B, which is different from CBM32 in reported alginate lyases. However, the FL enzyme exhibited lower activity than TM1 and TM2, which indicated an antisynergistic effect of CBMs. The synergy of CBMs linked by covalent bonds was first reported in 2001; such linkage produced 4–45-fold higher affinities for two serial CBM29 proteins than either of the separate CBM29 modules [16]. Up to now, there was little report of the antisynergistic effect of CBMs in carbohydrate active enzymes. The tandem CBMs and catalytic module might interfere with each other, resulting in the lower activity of full-length enzyme.

Compared with the non-CBM9 proteins TM1 and TM3, FL and TM2 showed higher activity in the alkaline environment. However, when experiments were performed under acidic conditions, the enzymatic activities of FL, TM1, and TM2 were clearly lower than that of TM3. These results indicated that both CBM9 and CBM32 promoted adaption of the PL7 catalytic module towards an alkaline environment, which might result from the enhanced binding of alginate by CBMs in alkaline environment. The enzymes, including the CBMs and catalytic module, may not be folded in its native state under alkaline conditions. 

After storage at 30 °C for 1 h, the activity of TM3 was approximately 57%, much higher than that of TM2. This was similar to the results reported for Aly2 by Peng et al. [22]. Notably, after the FL protein had been stored at 60 °C for 1 h, it retained approximately 20% activity and still had 5% activity after incubation at 80 °C for 1 h. We identified an unusual synergy in thermostability between CBM9 and CBM32, which had never been reported previously. The precise mechanism underlying this observation remains unclear and requires further research, which should particularly aim to identify the amino acids responsible for such synergy. We believe that this characteristic could be used to direct the evolution of thermostability in alginate lyases. 

CBM32 was previously shown to directly participate in the substrate recognition required for catalytic action [23]; this was consistent with the current substrate specificity results for the TM1 recombinant protein. We also verified that both CBM9 and CBM32 could enhance the recognition of alginate lyase for polyG and increase the DP of the final degradation products; this may result from the substrate preference of CBM9 and CBM32, since different CBMs could recognize and bind to different carbohydrate. Future research should explore specific binding sites and elucidate the molecular mechanism. The end products of alginate degraded by full-length TsAly7B and three mutants respectively were unsaturated disaccharides, trisaccharides, and tetrasaccharides but not monosaccharides. This result indicated that these enzymes degraded alginate in an endolytic manner, since exo-type alginate lyases degrade alginate to produce monosaccharides. Both CBM9 and CBM32 did not alter the action manner of TsAly7B.

In conclusion, CBM9 was shown to enhance the activity of the catalytic module in an alkaline environment. CBM32 was shown to increase the thermal stability and to withstand acidic and alkaline environments. These two CBMs also altered the substrate recognition and the DP of the final degradation products. In addition, we showed that the co-existence of CBM9 and CBM32 limited their independent ability to improve the catalytic activity of the PL7 catalytic module. This discovery provides new insights for the rational design of enzymes by fusion between CBMs and catalytic modules.

## 4. Materials and Methods

### 4.1. Bacterial Strains, Plasmids, and Chemicals

Marine bacterium *Thalassomonas* sp. LD5 was stored in our laboratory and in the China Center for Type Culture Collection (CCTCC; AB2017194). *E. coli* strains DH5α and BL21(DE3) (TaKaRa, Dalian, China) were cultured at 37 °C in Luria-Bertani (LB) broth or on LB agar plates containing kanamycin (30 μg/mL) when relevant. Plasmid pET-28a (+) (Novagen, Beijing, China) was used to express the recombinant proteins. T4 DNA ligase and PrimeSTAR DNA polymerase were purchased from TaKaRa (Dalian, China). Genomic DNA was extracted from *Thalassomonas* sp. LD5 using a commercial DNA purification kit (Thermo Scientific, Beijing, China). Sodium alginate was from Bright Moon Seaweed Group (Qingdao, China). PolyM and polyG (purity: about 95%) were from Qingdao BZ Oligo Biotech Co., Ltd. (Qingdao, China).

### 4.2. Cloning and Sequence Analysis of TsAly7B

PCR primers were designed using the genomic sequence of *Thalassomonas* sp. LD5 and used to amplify the FL sequence of TsAly7B and a series of truncated mutants (TM1, TM2, and TM3). The primer sequences are shown in Table 4. To express the FL, TM1, and TM3 proteins, the PCR products were digested with *Nco* I and *Xho* I, then ligated into the similarly digested pET-28a (+) expression vector. TM2 was designed to delete the CBM32 from Ile179 to Asn337 based on FL sequence. Two fragments were obtained by PCR using primer pairs TsAly7B-TM2-1-F/R and TsAly7B-TM2-2-F/R, digested with *Bam*H I, and ligated by T4 DNA ligase. PCR was performed to amplify TM2 sequence using the resulted fragment as template and TsAly7B-TM2-1-F and TsAly7B-TM2-2-R as the primers. The PCR product was digested with *Nco* I and *Xho* I, then ligated into the similarly digested pET-28a (+) expression vector to express TM2. The Compute pI/Mw tool (https://web.expasy.org/compute_pi/) was applied to calculate the theoretical molecular weight and pI. Clustal Omega Web (https://www.ebi.ac.uk/Tools/msa/clustalo/) was used to obtain multiple sequence alignments. Signal peptides were predicted by the SignalP 5.0 server (http://www.cbs.dtu.dk/services/SignalP/). CD-search was performed using NCBI (https://www.ncbi.nlm.nih.gov/Structure/cdd/wrpsb.cgi/).

### 4.3. Expression of TsAly7B and Three Truncated Mutants

*E. coli* BL21(DE3) strain, which had been transformed with the four recombinant plasmids, respectively, was cultured at 180 rpm and 37 °C for 3 h until the optical density at 600 nm reached 0.4. Then, the cultures were transferred into an incubator at 18 °C, with shaking at 100 rpm, for an additional 24 h with 0.1 mM isopropyl-β-thiogalactoside (IPTG) for FL and TM1, and 0.2 mM IPTG for TM2 and TM3. Subsequently, the cells were disrupted using a JN-mini-10C cell cracker (JNBIO, Guangzhou, China) and then centrifuged at 12000 rpm for 20 min to separate cell debris and unbroken cells. Recombinant proteins were then purified from the supernatants using a HisTrap HP column (GE Healthcare, Boston, USA). The binding buffer was 20 mM Na_2_HPO_4_-NaH_2_PO_4_ (pH 7.3) with 500 mM NaCl, and the elution buffer was 20 mM Na_2_HPO_4_-NaH_2_PO_4_ (pH 7.3) with 500 mM NaCl and 100 mM imidazole. The molecular weight and purity of the recombinant proteins were determined by sodium dodecyl sulfate polyacrylamide gel electrophoresis (SDS-PAGE) on a 10% (*w/v*) resolving gel. The software Phoretix was used to calculate the molecular weight and purity.

### 4.4. Alginate Lyase Activity Assay

Alginate lyases degrade alginate by the β-elimination mechanism and generate a double bond between C4 and C5 of the sugar ring at the newly formed non-reducing end that can be monitored as a change in absorbance at 235 nm. Enzyme activity was determined with 100 μL of enzyme solution and 900 μL of substrate solution (0.3% (*w/v*) sodium alginate, 20 mM phosphate buffer, 500 mM NaCl, pH 7.3) at 30 °C for 10 min. Absorbance measurements were taken by UH5300 UV-visible spectrophotometer (HITACHI, Japan). One unit (U) was defined as the amount of enzyme required to increase the absorbance at 235 nm by 0.1 per min [13]. In each experiment, pre-experiments were done to adjust the protein concentration and keep the value within the credible A_235_ range from 0.2 to 1.5.

### 4.5. Biochemical Characterization of Recombinant TsAly7B and Three Truncated Mutants

The optimal reaction temperature for the four recombinant proteins was determined by testing the enzymatic activity at 0–50 °C. To determine the optimal reaction pH, we generated four reaction buffers with different pH values: 50 mM Na_2_HPO_4_-citric acid (pH 3.0–8.0), 50 mM Na_2_HPO_4_-NaH_2_PO_4_ (pH 6.0–8.6), Tris-HCl (pH 7.0–9.0), and 50 mM glycine-NaOH (pH 8.6–10.6). With regards to thermostability, we stored the enzymes for 1 h at temperatures ranging from 0 to 50 °C for FL, and from 0 to 80 °C for TM1, TM2, and TM3; we then determined the residual activity. We stored the recombinant proteins at 4 °C for 6 h at different pH values, then measured the residual activity to determine the pH stability. We also investigated the effects of metal ions and chelators on the activity of TsAly7B and the three truncated mutants. The highest activity value was defined as 100% in these experiments. PolyM and polyG were used as reaction substrates in order to identify substrate specificities. All experiments were performed in triplicate; enzymatic activity is presented as mean ± standard deviation.

### 4.6. End Products Analyzed by TsAly7B and Three Truncated Mutants

We analyzed the end products resulting from enzymatic degradation of alginate by recombinant FL, TM1, TM2, and TM3. To obtain the end products, the recombinant enzyme (25 U) was added into 10 mL of 0.3% (*w/v*) alginate solution and incubated under optimal reaction conditions for 12 h. At 3, 6 and 9 h, another 25 U enzyme was added to the reaction mixture. We identified the final degradation products by fast protein liquid chromatography (FPLC) with a Superdex Peptide 10/300 Gel Filtration Column (GE Healthcare, Boston, USA), in which the mobile phase was 0.20 M NH_4_HCO_3_, at a flow rate of 0.4 ml/min. The eluted fractions were analyzed at a wavelength of 235 nm using an ultraviolet detection system. The mixture of unsaturated disaccharides, trisaccharides, and tetrasaccharides was used as the marker, and the total uronic acid concentration was 0.2% (*w/v*).

## Figures and Tables

**Table 1:** Effects of reagents on activities of TsAly7B and truncated mutants.

Reagents Added	Concentration (mM)	Relative Activity (%)			
		TsAly7B-FL	TsAly7B-TM1	TsAly7B-TM2	TsAly7B-TM3
None	-	100	100	100	100
NH_4_Cl	1	108.13 ± 3.56	102.04 ± 4.39	104.4 ± 12.19	102.31 ± 2.41
LiCl	1	105.58 ± 9.71	99.78 ± 4.37	77.14 ± 9.68	95.81 ± 9.31
KCl	1	104.28 ± 5.86	96.6 ± 3.53	107.47 ± 5.3	103.05 ± 2.94
MgCl_2_	1	100.39 ± 2.37	100.7 ± 7.84	83.19 ± 3.92	100.57 ± 5.58
CaCl_2_	1	114.36 ± 6.95	89.91 ± 12.71	100.82 ± 4.81	105.11 ± 10
MnCl_2_	1	89.31 ± 7.92	93.31 ± 24.01	84.89 ± 3.58	50.47 ± 3.85
BaCl_2_	1	91.29 ± 16.32	86.71 ± 10.09	50.34 ± 3.6	83.76 ± 7.6
EDTA	1	3.29 ± 0.58	1.1 ± 0.77	4.61 ± 3.59	2.26 ± 1.72
SDS	1	100.9 ± 3.19	89.86 ± 3.14	79.15 ± 3.94	90.24 ± 7.2

The relative activity of 100% was determined at optimal pH and temperature in the presence of 0.5 M NaCl.

**Table 2:** The specific activity of TsAly7B and truncated mutants.

Protein	Specific Activity (U/mg)	Molecular Weight (kDa)	Specific Activity (U/nmol)
TsAly7B-FL	488.8 ± 12.4	64.79	31.6 ± 0.8
TsAly7B-TM1	869.1 ± 9.1	51.35	44.6 ± 0.5
TsAly7B-TM2	1147.2 ± 19.7	52.66	60.4 ± 1.0
TsAly7B-TM3	238.4 ± 5.6	33.78	8.0 ± 0.2

Values were determined at the optimal pH and temperature in the presence of 0.5 M NaCl.

**Table 3:** The substrate specificity of TsAly7B and truncated mutants.

	Relative Activity (%)			
Substrate	FL	TM1	TM2	TM3
Alginate	100	100	100	100
PolyM	110.93 ± 3.23	96.61 ± 12.46	97.08 ± 2.43	107.38 ± 28.33
PolyG	91.29 ± 4.6	81.47 ± 18.13	103.21 ± 5.81	43.05 ± 11.62

The relative activity of 100% was determined at optimal pH and temperature in the presence of 0.5 M NaCl.

**Table 4:** Primers used in this study.

Primers	Sequence (5′ to 3′)	Usage
Expression of TsAly7B and truncated proteins		
TsAly7B-FL-F	CATG*CCATGG*GCTTGATTAATAATAAATTAAAAAAATGTG	Expression of TsAly7B-FL
TsAly7B-FL-R	CCG*CTCGAG*AGGGTTATAACCCGTATGTGA	
TsAly7B-TM1-F	CATG*CCATGG*GCATCGATAATGGTACTCATGATG	Expression of TsAly7B-TM1
TsAly7B-TM1-R	CCG*CTCGAG*AGGGTTATAACCCGTATGTGA	
TsAly7B-TM2-1-F	CATG*CCATGG*GCTTGATTAATAATAAATTAAAAAAATGTG	Expression of TsAly7B-TM2
TsAly7B-TM2-1-R	CG*GGATCC*GGCATTCACAATGGTTTGCG	
TsAly7B-TM2-2-F	CG*GGATCC*ACGAAGATCAATGGCTGTACT	
TsAly7B-TM2-2-R	CCG*CTCGAG*AGGGTTATAACCCGTATGTGA	
TsAly7B-TM3-F	CATG*CCATGG*GCCCGCCTTCTGGTAATTTCG	Expression of TsAly7B-TM3
TsAly7B-TM3-R	CCG*CTCGAG*AGGGTTATAACCCGTATGTGA	
